# A curated binary pattern multitarget dataset of focused ATP-binding cassette transporter inhibitors

**DOI:** 10.1038/s41597-022-01506-z

**Published:** 2022-07-26

**Authors:** Sven Marcel Stefan, Patric Jan Jansson, Jens Pahnke, Vigneshwaran Namasivayam

**Affiliations:** 1 Department of Pathology, Section of Neuropathology, Translational Neurodegeneration Research and Neuropathology Lab (www.pahnkelab.eu), University of Oslo and Oslo University Hospital, Sognsvannsveien 20, 0372 Oslo, Norway; 2 Department of Pharmaceutical and Cellbiological Chemistry, Pharmaceutical Institute, University of Bonn, An der Immenburg 4, 53121 Bonn, Germany; 3 Cancer Drug Resistance & Stem Cell Program, School of Medical Science, Faculty of Medicine and Health, The University of Sydney, Camperdown, NSW 2006 Australia; 4 LIED, Pahnke Lab, University of Lübeck and University Medical Center Schleswig-Holstein, Ratzeburger Allee 160, 23538 Lübeck, Germany; 5 Bill Walsh Translational Cancer Research Laboratory, Kolling Institute, Faculty of Medicine and Health, The University of Sydney, St. Leonards, NSW 2065 Australia; 6 Department of Pharmacology, Faculty of Medicine, University of Latvia, Jelgavas iela 4, 1004 Rīga, Latvia; 7 Department of Neurobiology, The Georg S. Wise Faculty of Life Sciences, Tel Aviv University, P.O. Box 39040, 6997801 Tel Aviv, Israel

**Keywords:** Drug discovery and development, Chemical biology

## Abstract

Multitarget datasets that correlate bioactivity landscapes of small-molecules toward different related or unrelated pharmacological targets are crucial for novel drug design and discovery. ATP-binding cassette (ABC) transporters are critical membrane-bound transport proteins that impact drug and metabolite distribution in human disease as well as disease diagnosis and therapy. Molecular-structural patterns are of the highest importance for the drug discovery process as demonstrated by the novel drug discovery tool ‘computer-aided pattern analysis’ (‘C@PA’). Here, we report a multitarget dataset of 1,167 ABC transporter inhibitors analyzed for 604 molecular substructures in a statistical binary pattern distribution scheme. This binary pattern multitarget dataset (ABC_BPMDS) can be utilized for various areas. These areas include the intended design of (i) polypharmacological agents, (ii) highly potent and selective ABC transporter-targeting agents, but also (iii) agents that avoid clearance by the focused ABC transporters [*e.g*., at the blood-brain barrier (BBB)]. The information provided will not only facilitate novel drug prediction and discovery of ABC transporter-targeting agents, but also drug design in general in terms of pharmacokinetics and pharmacodynamics.

## Abstract (summary)

**Table:** 

Measurement(s)	Influx • Efflux • Tracer • Transport velocity
Technology Type(s)	Fluorometry • Radioactivity • Plate reader • Flow cytometer • Tracer distribution
Factor Type(s)	half-maximal inhibition concentration
Sample Characteristic - Organism	Homo sapiens
Sample Characteristic - Environment	cell culture
Sample Characteristic - Location	Kingdom of Norway • Germany • Australia • Latvia

## Background & Summary

The superfamily of ABC transporters is of highest importance in terms of novel drug discovery, design, and development. ABC transporters are ubiquitously present in the human body^1–4^, and their (co-)expression has broad implications in human diseases. These diseases include prevalent [*e.g*., Alzheimer’s disease (AD)^5,6^, atherosclerosis^7^, or cancer^1,3,6,8^] and orphan [*e.g*., Tangier disease (ABCA1)^9^, Stargardt’s disease (ABCA4)^10^, harlequin ichthyosis (ABCA12)^11^, pseudoxanthoma elasticum (ABCC6)^12^, or adrenoleukodystrophy (ABCD1)^13^] pathological conditions. Together with tight-junction proteins, these membrane-bound efflux pumps are the backbone of systemic barrier formation^14,15^. Their localization at blood-tissue barriers impacts metabolite distribution and drug delivery, and hence, disease progress, treatment, and therapy^15–19^. Determinants that establish a correlation between the molecular structure of a small-molecule (drug) and its interaction with ABC transporters is key for the development of novel, safe, systemically applicable, and target-oriented (selective) drugs.

These determinants include descriptors that conserve certain physicochemical features of the small-molecules of interest, such as the calculated octanol-water partition coefficient (CLogP), molecular weight (MW), molar refractivity (MR), or topological polar surface area (TPSA), but also the number of hydrogen bond (H-bond) donors, H-bond acceptors, or rotatable bonds^5^. Other than that, more complex attributes can be summarized in fingerprints that represent certain molecular features of the small-molecule in a binary code (*e.g*., feature-, path-, and radial-fingerprints^20–22^). Unfortunately, comprehensive binary datasets do not exist for ABC transporters. However, the knowledge about such binary fingerprints could facilitate the development of (i) drugs that avoid clearance mediated by ABC transporters [*e.g*., targeting the BBB to treat central nervous system-(CNS)-related diseases^23^]; (ii) agents targeting ABC transporters to study their expression and/or function with state-of-the-art imaging techniques [*e.g*., by positron emission tomography (PET)^16^]; (iii) drugs that selectively target well-studied ABC transporters in human diseases (*e.g*., cancer^1,3,4,6,8^); (iv) broad-spectrum drugs that target several ABC transporters to ameliorate/cure an ABC transporter-associated pathological condition^24^; (v) polypharmacological agents to target and study particularly less- and under-studied ABC transporters by a multitargeting approach^7,25–27^; or (vi) combined/extended fingerprints to create high-quality compound collections that would provide a starting point of polypharmacology-focused virtual screenings^7^.

In the present work, we combined the concepts of the multitarget dataset^7,27^ and the binary distribution of substructures^7^. The latest version of the multitarget dataset contains 1,167 compounds that were evaluated against the well-studied ABC transporters ABCB1, ABCC1, and ABCG2. A large substructure catalog was created, containing in total 604 active (= present) substructures within these 1,167 compounds of the updated multitarget dataset. The new binary pattern multitarget dataset (ABC_BPMDS) is freely available under the http://www.zenodo.org^28^ URL as well as the http://www.panabc.info website, and its use is free of charge.

## Methods

**Fig. 1:**
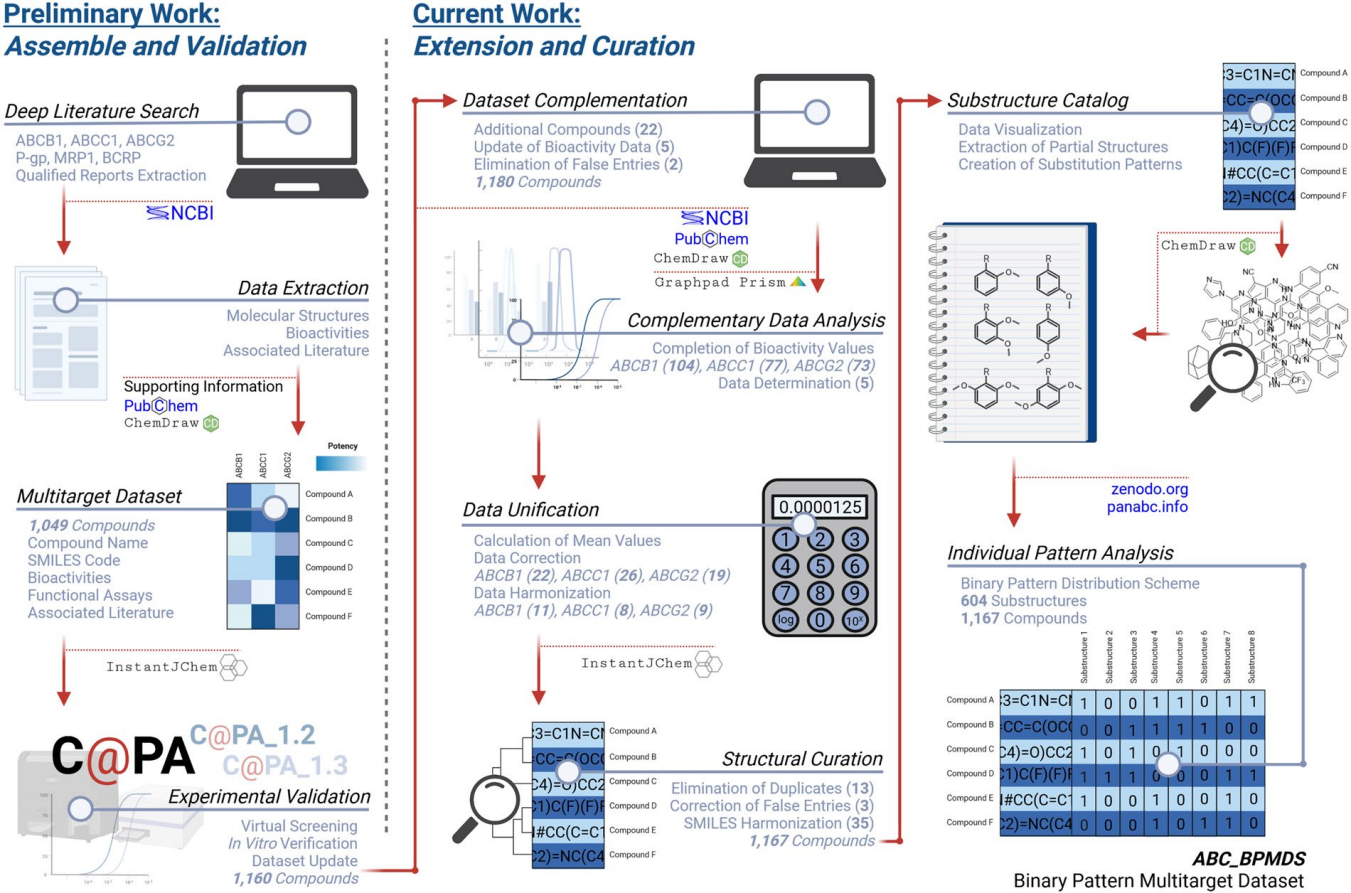
Depiction of the main workflow of assemble and validation as reported earlier in our preliminary work^27^, as well as the main steps of data extension and curation as part of the current work to generate the ABC_BPMDS. This graphic was created with BioRender.com (https://biorender.com).

The generation of the ABC_BPMDS was a four-step process: (i) deep literature search including the selection of qualified reports, resulting in the exquisite compilation of the original multitarget dataset as reported earlier^27^ [including updates in our former^7^ and the present work (see below)]; (ii) manual curation of the given data, in particular: (a) calculation of bioactivity values for estimated bioactivity data and data determination, (b) unification and harmonization of bioactivity data, as well as (c) comparison, curation, and harmonization of molecular-structural data (SMILES codes); (iii) generation of a substructure catalog, in particular: (a) visual inspection of the 1,167 molecules of the updated multitarget dataset, (b) extraction of partial structures, (c) creation and extension of substitution patterns, as well as (d) screening of the multitarget dataset for these substructures, discovering 604 active substructures; and (iv) individual pattern analysis^7^ for uncovering the statistical distribution of these 604 active substructures amongst the 1,167 compounds of the multitarget dataset. The following sections will provide a detailed description on how the final ABC_BPMDS was assembled. Figure 1 provides an overview of the taken steps.

### Literature Collection of the Original Dataset

#### Qualified Reports

A deep literature search was the first step to compile the original multitarget dataset, which has been reported in detail before^7,27^. The National Center for Biotechnological Information (NCBI; https://www.ncbi.nlm.nih.gov)^29^ was used to search for qualified reports applying the keywords (i) ‘ABCB1’, (ii) ‘ABCC1’, (iii) ‘ABCG2’, (iv) ‘P-gp’, (v) ‘MRP1’, and (vi) ‘BCRP’. The keywords were used in all possible combinations to extract the maximal yield in reports. In addition to the genuine database search, the reference sections of the found reports were searched for potential additional literature to extract further qualified information.

#### Compounds

Compounds were considered only if they had been evaluated against all three focused targets, ABCB1, ABCC1, and ABCG2, including inactive compounds as well as selective, dual, and triple inhibitors. This information could be provided either in one single report (*e.g*., in case of the standard ABCG2 inhibitor Ko143^30^) or in several individual reports [*e.g*., in case of the standard ABCC1 inhibitor verlukast (MK571)^31–36^]. The molecular structures of qualified compounds were collected as SMILES codes. These were obtained either from (i) supplementary information of the respective report; (ii) the PubChem database (https://pubchem.ncbi.nlm.nih.gov)^37^ [*e.g*., in case of known drugs and drug-like compounds, such as the standard inhibitors verapamil (ABCB1), cyclosporine A (ABCB1 and ABCC1), verlukast (ABCC1), or Ko143 (ABCG2)]; or (iii) manual drawing according to the 2D representations as outlined in the respective report using ChemDraw Pro version 20.1.1.125. Isomeric SMILES were considered where applicable. SMILES codes that encoded aromatic substructures with lower-case letters in certain reports^38,39^ were unified according to the upper-case description scheme (structural curation)^7^.

#### Assays

Only functional assays were considered using either fluorescence labeling or radionuclide detection applying either living (selected or transfected) cells or membrane vesicles with reconstituted transporters. ATPase assays were not considered because ATPase activity and transporter inhibition may not be directly connected to each other. MDR reversal assay data was not considered because of the complexity of the involved processes and the fact that the triggered response(s) may not only be caused by ABC transporter inhibition. Table 1 provides an exhaustive list of functional tracers (and substrates) that were used to assess the 1,167 compounds of the ABC_BPMDS against ABCB1, ABCC1, and ABCG2. Table 2 summarizes all used host systems (cell lines and membrane vesicles) used for the evaluation of the 1,167 compounds against ABCB1, ABCC1, and ABCG2.

**Table 1:** An exhaustive list of functional tracers that were used to functionally assess the 1,167 compounds of the ABC_BPMDS against ABCB1, ABCC1, and ABCG2^7,27^.

The assessment of the corresponding transporter by the respective tracer is indicated by a black box. The provided references are examples in which details in terms of the stated assays can be found^7,27,47,55,84–101^.

**Table 2:** An exhaustive list of transporter host systems that were used to functionally assess the 1,167 compounds of the ABC_BPMDS against the well-studied ABC transporters ABCB1, ABCC1, and ABCG2^7,27^.

The assessment of the corresponding transporter by the respective host system is indicated by a black box. Regarding selected cells, the used cytotoxic agent is indicated under the respective transporter, and the cell subline abbreviation is given in brackets. The provided references are examples in which details in terms of the stated cell lines can be found^7,38,42,48,52,84,89,93,95,97–114^.

#### Bioactivity

The bioactivities (IC_50_ values) of the compounds were extracted from either (i) tables of the respective reports (including supplementary information); or (ii) screening figures with relative inhibition (I_rel_) values (%) compared to a standard (I_max_; 100%). In the latter case, the IC_50_ values were estimated (either span or >, ≥, <, ~) in the previous multitarget dataset^7,27^.

### Data Curation – Bioactivity Data

#### Dataset Update and Complementation

New reports particularly from 2021 and 2022 were taken into consideration to update the dataset with compounds that were evaluated against the three transporters ABCB1, ABCC1, and ABCG2. In total, 22 new compounds were included into the list of qualified compounds^7,40–42^. In addition, we focused an extended literature search, particularly of known standard inhibitors of ABCB1, ABCC1, and ABCG2 to obtain bioactivities with less mathematical uncertainty which also align well with our empirical experience in the laboratory. These compounds included verapamil (ABCB1^43^), cyclosporine A (ABCB1^41,43–46^ and ABCC1^31,44–46^), verlukast (ABCC1^31–36^), and Ko143 (ABCG2^41,45^). As a side note, the additional literature search also resulted in an update of bioactivity data of the natural compound piperine^47^. In the curation process to complement bioactivity values, we found that two compounds were erroneously included into the dataset (apatinib^48^ and ceritinib^49^). Both were not evaluated against ABCC1, and therefore, did not qualify for this dataset and were therefore removed.

#### Complementary Data Analysis

The bioactivity of several inhibitors could only be described as an estimation (either described as span, marked as ‘active’, or annotated with ‘>’, ‘≥’, ‘<’, ‘~’ in the previous dataset^7,27^). However, to allow for the use of the entire dataset in mathematical and computational operations, we sought to allocate defined bioactivity values to these compounds. Hence, the individual reports were analyzed and the given indications of bioactivity [*e.g*., screening figures, flow-cytometry histograms, or tables with bioactivity values other than IC_50_ values (*e.g*., percentages)] were taken into consideration for further data analysis. The specific bioactivity value (*e.g*., percentage inhibition) was extracted and correlated to the used compound concentration. By using GraphPad Prism version 8.4.0 applying the three-parameter logistic equation with a fixed Hill slope (=1.0), IC_50_ values were calculated and listed in the new multitarget dataset. A detailed curation protocol is provided on https://www.zenodo.org^50^ as well as he http://www.panabc.info website, and the related GraphPad Prism file containing the concentration-effect curves can be accessed without restrictions. In total, the bioactivity data of 104, 77, and 73 ABCB1, ABCC1, and ABCG2 inhibitors, respectively, have been calculated and complemented.

#### Data Determination

The bioactivities of five compounds [ayanin^51^, retusin^51^ (flavone derivative 12^52^), dihydrodibenzoazepine derivative 4i^53^, dregamine derivative 2^54^, and tabernaemontanine derivative 22^54^] had to be determined without mathematical operations. The IC_50_ values of ayanin and retusin were stated as ‘>50 µM’ in the original report^51^. Usually, these kinds of statements (*e.g*., ‘>50 µM’, ‘>100’, ‘inactive’, *etc*) led to the allocation of such compounds into the ‘inactive’ category (arbitrary IC_50_ value of 2000 µM in the ABC_BPMDS). However, the authors of the respective publication stated that ayanin and retusin had some (weak) inhibitory activity^51^. Therefore, we decided to allocate an arbitrary value of 100 µM to these compounds to acknowledge their minor inhibitory potential against ABCC1. Dihydrodibenzoazepine derivative 4i^53^, dregamine derivative 2^54^, and tabernaemontanine derivative 22^54^, on the other hand, reached over 100% inhibition at concentrations of 2.50 µM, 20.0 µM, and 20.0 µM, respectively. Unfortunately, these were the only indications of bioactivity by the authors of the original reports^53,54^. Hence, we decided to allocate arbitrary values of 0.999 µM^53^, 4.99 µM^54^, and 4.99 µM^54^, respectively, to acknowledge their potentially (very) high inhibitory power against ABCB1 as well as ABCG2 considering the effect-concentrations used in the original reports. These arbitrary IC_50_ values have been chosen since sub-classifications of bioactivity classes according to bioactivity thresholds (*e.g*., 1 and 5 µM) provided a better prediction in our previous works^7^.

#### Data Unification

Several compounds were evaluated in multiple assays, *e.g*., the mentioned standard inhibitors of ABCB1, ABCC1, and ABCG2. However, to allocate one bioactivity value to one compound, a unification process was necessary. As IC_50_ values do not follow a normal distribution, the multiple IC_50_ values associated with one compound were subject to a three-step mathematical operation: (i) logarithmization of the IC_50_ values; (ii) calculation of the mean; and (iii) delogarithmization of the log(IC_50_)-mean value. The resultant mean value was allocated to the respective compound. It shall be noted that the bioactivities of the compounds curcumin I-III (ABCC1)^55^ and gefitinib (ABCB1 and ABCC1)^56^ were only given as a span in the original reports^55,56^, and hence, the mean of the respective span was taken for further operations. In total, 60, 48, and 209 ABCB1, ABCC1, and ABCG2 inhibitors have been given a new bioactivity value by these operations compared to the previous multitarget dataset^7,27^.

#### Data Correction and Harmonization

Through the complementary analysis process, several bioactivity values were corrected. This applied for compounds that were falsely marked as ‘inactive’ in the previous multitarget dataset (ABCB1: 22 compounds; ABCC1: 26 compounds; ABCG2: 19 compounds)^7,27^. Lastly, all bioactivity values of the ABC_BPMDS were harmonized according to a number of three significant digits. This harmonization resulted in a standardized format of presentation: (i) ‘XXX0 µM’; (ii) ‘XXX µM; (iii) XX.X µM; (iv) X.XX µM; (v) 0.XXX µM; and (vi) 0.0XX (X = any numeric value between 1–9). Here, 11, 8, and 9 ABCB1, ABCC1, and ABCG2 values have been changed compared to the previous multitarget dataset^7,27^.

### Data Curation – Molecular-structural Data

The 1,167 compounds of the ABC_BPMDS were portrayed as canonical or isomeric SMILES codes as derived from the (i) respective report, (ii) PubChem database (https://pubchem.ncbi.nlm.nih.gov), or (iii) SMILES generation tool of ChemDraw Pro version 20.1.1.125. All smiles were compared to each other to identify duplicates by using InstantJChem version 21.13.0. Through this individual cross-check of the molecular-structural data, 13 compounds were discovered as duplicates^46,51,56–59^ and their bioactivity values were merged with the original bioactivity data of the particular compound^52,59–62^. In addition, three compounds were identified to be incorrect in terms of their molecular structure and have been corrected in the dataset^46,57,63^.

### Binary Pattern Generation

#### Background

In contrast to common molecular fingerprints for similarity-based virtual screenings^20,64^, the very recently reported novel drug discovery tool ‘computer-aided pattern analysis’ (‘C@PA’) identified that defined (=non-substituted) hydrogens and their positioning is particularly important in terms of the differentiation between selective and multitarget inhibition of ABC transporters^7,26,27^. Although certain fingerprints indeed consider polar hydrogens^21,22^, C@PA particularly discovered non-polar hydrogens with critical discriminatory potential in the virtual screening process^7,26,27^. However, the original C@PA worked with a very preliminary and limited dataset of 308 substructures which were compiled after multitarget dataset visualization and literature consideration^65^, of which only 162 substructures were active in the multitarget dataset of, at the time of the study, 1,049 compounds^27^.

#### Substructure Visualization, Identification, and Extension

For the development of a complete, detailed, and novel (multitarget) fingerprint, which may also universally be used in (multitarget) virtual screening approaches, the 1,167 compounds of the updated multitarget dataset were visualized using ChemDraw Pro version 20.1.1.125, and substructures were identified and extracted. The extracted substructures [*e.g*., single-standing/centered (hetero-)aromatic rings, condensed (hetero-)aromatic rings, (un)saturated side chains, extremities, and non-aromatic (hetero-)cycles, *etc*.] were derivatized by applying a heavy atom substitution scheme as already reported earlier^26^ (scaffold fragmentation and substructure hopping). Especially the presence and positioning of (non-polar) hydrogens in the sense of a proton/non-proton pattern scheme was stressed. These measures increased the quantity of substructural properties covered by the intended fingerprint. In addition, alternative datasets of ABC transporter modulators^5^ and modes of action (particularly ABC transporter activators)^6,8^ have been considered to gain complementary knowledge about potentially active substructures. The resultant substructures were subsequently searched in the 1,167 compounds (loaded as.csv file) using the query search function of InstantJChem version 21.13.0 and, if present, listed in the substructure catalog. As a result, a catalog of 604 active substructures has been assembled.

#### Individual Pattern Analysis^7^

In a final step, the multitarget dataset of 1,167 compounds was statistically analyzed for the listed 604 substructures of the substructure catalog. Here, the resultant list of hit molecules per substructure derived from the query search function of InstantJChem version 21.13.0 was saved and compared to the original list, translating the entry differences into a binary code [1 = substructure present (active substructure); 0 = substructure not present (inactive substructure)]. A binary pattern distribution scheme resulted which constituted the final ABC_BPMDS. It shall be taken note that the number of the very same substructure within the same compound was irrelevant; the presence (numeric value = 1) of the substructure was not an expression of how often the respective substructure appeared within the compound.

## Data Records

The ABC_BPMDS is freely available in an .xlsx format under the http://www.zenodo.org^28^ URL as well as the http://www.panabc.info website and its use is free of charge. The dataset consists of (i) an individual database identifier for each compound; (ii) the original name of the compounds according to the original report(s); (iii) the IUPAC nomenclature of each compound generated by using ChemDraw Pro version 20.1.1.125; (iii) The SMILES code obtained either from the (a) supporting information of the respective report, (b) PubChem database (https://pubchem.ncbi.nlm.nih.gov), or (c) manual drawing using ChemDraw Pro version 20.1.1.125; (iv) the physicochemical properties (a) CLogP, (b) calculated molecular water solubility (CLogS), (c) MW, (d) MR, (e) TPSA, (f) H-bond donors, (g) H-bond acceptors, (h) rotatable bonds, and (j) number of heavy atoms; (v) the associated bioactivity values expressed as (a) IC_50_ values [µM] against ABCB1, ABCC1, and ABCG2 presented in the standardized format of three significant digits as outlined above [10^log(mean)^], and (b) pIC_50_ values against ABCB1, ABCC1, and ABCG2; (vi) the binary code (active = 1; inactive = 0) for each of the 604 evaluated substructures of the substructure catalog including their (a) trivial name, (b) SMILES code, (c) number of defined hydrogens, (d) number of heavy atoms, (e) total hit count, and (f) individual substructure identifier. The substructures are sorted from most abundant (left) to most rare (right); and (vii) the PubMed (https://pubmed.ncbi.nlm.nih.gov) identifier (PMID) retrieved from NCBI (https://www.ncbi.nlm.nih.gov). In addition, a detailed curation protocol as well as an associated GraphPad Prism file can be found on https://www.zenodo.org^50^ as well as the http://www.panabc.info website.

## Technical Validation

### Compounds

The 1,167 compounds were portrayed as canonical or isomeric SMILES codes as derived from the respective report or the PubChem database (https://pubchem.ncbi.nlm.nih.gov) and imported into the MarvinSketch editor implemented in InstantJChem version 21.13.0. If the loaded SMILES code appeared as the intended original molecular representation according to the respective report or the PubChem database (https://pubchem.ncbi.nlm.nih.gov) without any errors, it was considered as valid.

### Bioactivity Space Validation

In total, 113 reports between 1994 and 2022 have been collected, resulting in a final number of 1,167 compounds that were evaluated against ABCB1, ABCC1, and ABCG2, including inactive compounds as well as selective, dual, and triple inhibitors. Amongst the 1,167 compounds are (i) 525 ABCB1 inhibitors, of which (a) 88 are selective ABCB1 inhibitors (no activity against ABCC1 and ABCG2; any given IC_50_ value), (b) 67 are potent ABCB1 inhibitors (IC_50_ values < 1 µM), and (c) 25 are selective and potent ABCB1 inhibitors; (ii) 344 ABCC1 inhibitors, of which (a) 61 are selective ABCC1 inhibitors (no activity against ABCB1 and ABCG2; any given IC_50_ value), (b) 45 are potent ABCC1 inhibitors (IC_50_ values < 1 µM), and (c) 11 are selective and potent ABCC1 inhibitors; (iii) 866 ABCG2 inhibitors, of which (a) 409 are selective ABCG2 inhibitors (no activity against ABCB1 and ABCC1; any given IC_50_ value), (b) 330 are potent ABCG2 inhibitors (IC_50_ values < 1 µM), and (c) 199 are selective and potent ABCG2 inhibitors.

**Fig. 2:**
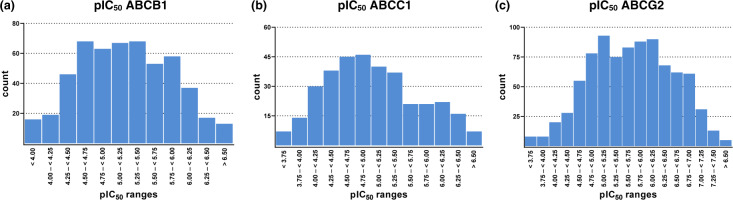
Distribution of bioactivity values (pIC_50_) of the 1,167 compounds of the ABC_BPMDS against ABCB1 (**a**), ABCC1 (**b**), and ABCG2 (**c**).

On the other hand, 38, 212, and 58 dual ABCB1/ABCC1, ABCB1/ABCG2, and ABCC1/ABCG2 inhibitors are present, respectively, of which 7, 99, and 13 can be considered as potent dual ABCB1/ABCC1, ABCB1/ABCG2, and ABCC1/ABCG2 inhibitors, respectively (IC_50_ < 10 µM). Finally, 187 triple ABCB1, ABCC1, and ABCG2 inhibitors can be defined, of which 54 can be considered as potent (IC_50_ < 10 µM; so-called ‘Class 7’ compounds^7,26,27^). Table 3 summarizes a survey of statistical parameters of the entire ABC_BPMDS as well as important sub-classes. Figure 2 depicts the distribution of the pIC_50_ values of ABCB1 (A), ABCC1 (B), and ABCG2 (C) inhibitors amongst the entire ABC_BPMDS, which followed in all three cases a Gaussian normal distribution.

**Table 3:** Statistical survey of the span as well as median and mean values of the bioactivity of the entire ABC_BPMDS as well as important sub-classes.

inhibitor class	count	IC_50_ span [µM]	pIC_50_ span	IC_50_ median [µM]	pIC_50_ median	IC_50_ mean [µM]	pIC_50_ mean
ABC_BPMDS	1,167	0.0153–1630	7.815–2.788	4.39	5.358	3.84	5.416
All ABCB1	525	0.0153–1460	7.815–2.836	6.37	5.196	6.32	5.199
All ABCC1	344	0.146–1630	6.836–2.788	11.2	4.951	9.26	5.033
All ABCG2	866	0.0234–405	7.631–3.393	1.95	5.710	2.00	5.698
Selective ABCB1	88	0.0153–708	7.815–3.150	2.51	5.599	3.41	5.467
Selective ABCC1	61	0.222–112	6.654–3.951	5.97	5.224	5.63	5.249
Selective ABCG2	409	0.0234–405	7.631–3.393	1.06	5.975	1.13	5.948
Dual ABCB1/ABCC1	38	0.289–180	6.539–3.745	20.4	4.692	15.2	4.819
Dual ABCC1/ABCG2	212	0.0255–333	7.593–3.478	4.43	5.354	3.85	5.415
Dual ABCC1/ABCG2	58	0.0988–163	7.005–3.788	10.1	4.996	6.92	5.160
Triple ABCB1/ABCC1/ABCG2	187	0.0475–1630	7.323–2.788	6.98	5.156	6.74	5.172

The pIC_50_ values have been calculated by using the negative decadic logarithm of the respective bioactivity value.

### Physicochemistry Space Validation

**Fig. 3:**
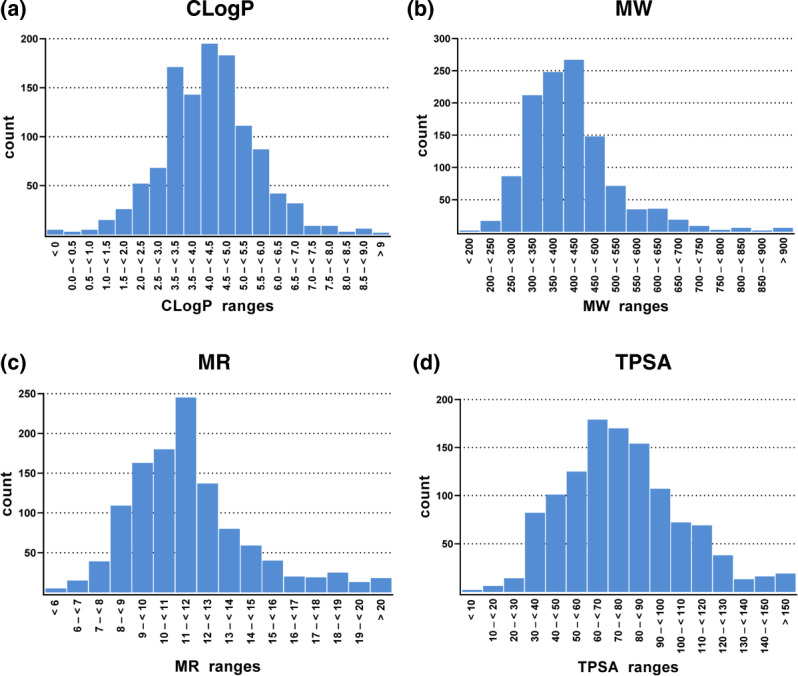
Distribution of the important physicochemical^115^ properties CLogP (**a**), MW (**b**), MR (**c**), and TPSA (**d**) amongst the 1,167 compounds of the ABC_BPMDS as determined by MOE version 2019.01.

Physicochemical properties shape not only the pharmacological profile of ABC transporter inhibitors^66–69^, but are also very often used as additional discriminators in virtual screening processes^7,26,27,38^. To prove that the 1,167 compounds of the ABC_BPMDS have a balanced distribution of physicochemical attributes, the ABC_BPMDS was analyzed for the CLogP, MW, MR, and TPSA using MOE version 2019.01. Figure 3 demonstrates that these physicochemical properties are normally distributed within the ABC_BPMDS comparable to other reported datasets^23,70^. Table 4 summarizes the median and mean values of CLogP, MW, MR, and TPSA of the entire ABC_BPMDS as well as important sub-classes. The median and mean values are well-aligned, which accounts for the equal distribution of values.

**Table 4:** Statistical survey of median and mean values of the important physicochemical properties CLogP, MW, MR, and TPSA amongst the entire ABC_BPMDS as well as important sub-classes as determined by MOE version 2019.01.

inhibitor class	count	CLogP median	CLogP mean	MW median	MW mean	MR median	MR mean	TPSA median	TPSA mean
ABC_BPMDS	1,167	4.33	4.26	403.39	418.38	11.27	11.73	73.86	77.87
All ABCB1	525	4.40	4.78	432.43	458.67	12.07	12.90	73.63	79.56
All ABCC1	344	3.91	3.88	420.44	442.92	11.72	12.37	76.10	84.82
All ABCG2	866	4.34	4.25	396.37	415.32	11.13	11.64	74.73	79.01
Selective ABCB1	88	5.22	5.47	452.11	481.94	13.08	13.70	61.42	65.98
Selective ABCC1	61	3.37	3.41	374.49	377.22	11.07	10.70	69.77	75.29
Selective ABCG2	409	4.38	4.35	372.38	381.58	10.39	10.67	73.86	75.14
Dual ABCB1/ABCC1	38	5.03	4.80	475.56	471.03	13.19	13.20	70.05	78.29
Dual ABCC1/ABCG2	212	4.42	4.52	420.23	434.62	11.86	12.32	75.69	75.83
Dual ABCC1/ABCG2	58	3.62	3.68	376.91	398.33	10.52	11.23	76.26	80.98
Triple ABCB1/ABCC1/ABCG2	187	3.95	3.91	432.44	472.47	11.91	13.10	79.44	90.45

### Molecular-Structure Space Validation

**Fig. 4:**
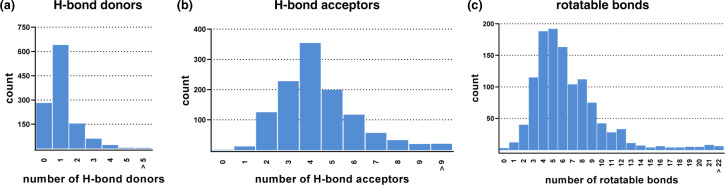
Distribution of H-bond donors (**a**), H-bond acceptors (**b**), and rotatable bonds (**c**) amongst the 1,167 compounds of the ABC_BPMDS as determined by MOE version 2019.01.

H-bonds and molecular flexibility are crucial aspects in terms of ligand-target interactions, especially for ABC transporters^71^. Hence, we analyzed the 1,167 compounds of the ABC_BPMDS for their number of H-bond donors, H-bond acceptors, and rotatable bonds. Figure 4 visualizes the found distributions amongst the entire ABC_BPMDS. Together with CLogP and MW, H-bond donors and acceptors play a major role in the drug-likeliness as defined by Lipinsky^72^, particularly influencing drug absorption, distribution, and permeation. Considering the ‘Lipinski rule of five’ (CLogP ≤ 5; MW ≤ 500; H-bond donors ≤ 5; H-bond acceptors ≤10), a large majority of compounds of the ABC_BPMDS fulfils these requirements. In particular, (i) 73.8% of compounds have CLogP values of ≤5, (ii) 84.0% of compounds have a MW of ≤500, (iii) 99.7% of compounds have ≤5 H-bond donors, and (iv) 98.6% of compounds have ≤10 H-bond acceptors. Table 5 summarizes the median and mean values of H-bond donors, H-bond acceptors, and rotatable bonds of the entire ABC_BPMDS as well as important sub-classes. Hence, the ABC_BPMDS contains suitable templates for future drug design and therapeutic development purposes, however, leaves also enough molecular-structural and physicochemical space for explorational analyses beyond the ‘Lipinski rule of five’ for the creation of inhomogeneous high-quality compound collections and compound libraries.

**Table 5:** Statistical survey of median and mean values of H-bond donors, H-bond acceptors, and rotatable bonds amongst the entire ABC_BPMDS as well as important sub-classes as determined by MOE version 2019.01.

inhibitor class	count	H-bond donors median	H-bond donors mean	H-bond acceptors median	H-bond acceptors mean	rotatable bonds median	rotatable bonds mean
ABC_BPMDS	1,167	1	1.12	4	4.43	6	6.50
All ABCB1	525	1	1.07	4	4.95	7	7.72
All ABCC1	344	1	1.28	4	4.87	6	6.91
All ABCG2	866	1	1.15	4	4.42	5	6.51
Selective ABCB1	88	1	0.75	4	4.70	8	7.70
Selective ABCC1	61	1	1.46	4	4.03	6	5.57
Selective ABCG2	409	1	1.14	4	3.79	5	5.35
Dual ABCB1/ABCC1	38	1	0.895	4	4.32	6	6.74
Dual ABCC1/ABCG2	212	1	0.986	4	4.78	7	7.87
Dual ABCC1/ABCG2	58	1	1.14	4	4.40	4.5	5.66
Triple ABCB1/ABCC1/ABCG2	187	1	1.35	4	5.40	6	7.76

## Usage Notes

### Status Quo

#### Practical Use

An easy-to-use sort function allows the user to discriminate the compounds regarding their bioactivities toward the targets, physicochemical properties, or molecular-structural features, but also in terms of the 604 different substructures. Hence, the user can retrieve the necessary binary pattern information for subsequent virtual screening and rational drug design approaches.

#### Special Considerations

The majority of the compounds was evaluated in proper full-blown concentration effect curves within the original report, providing either only one single IC_50_ or two IC_50_ values from different assays for biological validation, resulting mostly in minor standard deviations or standard errors. However, considering established reference compounds, many IC_50_ values have been reported that are not fully covered by the deep literature search. Moreover, these drugs and drug-like compounds were tested in various assays, and thus, their IC_50_ values vary in a greater span than of other compounds. In addition, data processing prior to the original publication varied from laboratory to laboratory [*e.g*., number of concentrations tested, manner of assay performance (non-standardized procedures), manner of data analysis (*e.g*., three- *vs* four-parameter logistic equation, relative *vs* absolute inhibition), data presentation (single-point screening graphic *vs* full-blown concentration effect curve, number of significant digits, in- or exclusion of standard deviation and/or standard error)] – contributing to a greater uncertainty of these particular data. Furthermore, the assays themselves that were considered for the ABC_BPMDS were various [*e.g*., influx *vs* efflux assay, fluorescence labeling *vs* radionuclide detection, manner of substrate (*e.g*., calcein AM *vs* mitoxantrone), selected cells *vs* transfected cells *vs* membrane vesicles) – contributing to a general variation in data that is hidden due to the fact that most compounds were only evaluated in one particular assessment system. These aspects should be considered when using the ABC_BPMDS, however, at the same time, it should be taken note that our previous work demonstrated the strength of substructural patterns based on the previous version of the ABC_BPMDS^7,26,27^. A list of compounds affected by these variations in assessment systems can be found in the curation protocol under the https://www.zenodo.org^50^ URL (10.5281/zenodo.6405752) or on the http://www.panabc.info web site.

### Future Perspective

#### Extension – New Compounds

The ABC_BPMDS provides the core application for extension to other, less- and under-studied ABC transporters. Particularly, the addressing of under-studied ABC transporters by multitarget agents poses a promising prospect for future drug discovery and development. Several compounds of the ABC_BPMDS have been demonstrated to address other ABC transporters as well^5,25,26^, such as benzbromarone^5,7,25–27^, cyclosporine A^5,7,25–27^, dipyridamole^7,27,73–77^, erlotinib^7,27,78,79^, imatinib^5,7,25–27^, nilotinib^7,27,78,80,81^, ritonavir^7,27,82^, verapamil^5,7,25–27^, and verlukast^5,7,25–27,33^. These ‘truly multitarget pan-ABC transporter inhibitors’^25^ are the primary focus for extension of the ABC_BPMDS, particularly with respect to their substructural elements that promote multitargeting. On the other hand, the addition of multitarget agents that are not part of the ABC_BPMDS will contribute valuable input to the polypharmacological space as charted by the future ABC_BPMDS_1.2.

#### Extension – New Substructures (‘ABC_BPMDS_1.2’)

The substructural elements of the mentioned truly multitarget pan-ABC transporter inhibitors include 4-anilinopyrimidine^7,27^, benzyl^7^, cyano^7,27^, 3,4-dimethoxyphenyl^7^, fluorine^7,27^, furan^7,26^, ethylene diamine^7^, ethylene hydroxy^7^, hydroxy^7^, isopropyl^7,27^, methylene hydroxy^7^, phenethyl^7^, piperazine^7,27^, pyrimidine^7,26^, quinazoline^7,27^, thiazlole^7,26^, and thioether^7^. These and other substructures will be re-evaluated with respect to true multitargeting, and thus, receive a differential value dependent on the purpose of the subsequent studies. Furthermore, the addition of multitarget agents that are not part of the ABC_BPMDS will contribute valuable input to the substructure catalog, extending the substructural output of the future ABC_BPMDS_1.2. Specifically, this information beyond known multitarget fingerprints will enable the exploration and exploitation of under-studied ABC transporters as potential drug targets of the future.

#### Extension – New Modes and Targets

Particularly, the inclusion of, for example, different modes of modulation (*e.g*., activation), bioactivity measurements [*e.g*., *in vitro* (ATPase assays or MDR reversal assays), *in silico* binding mode analyses (*e.g*., molecular docking or molecular dynamics simulations), or structural information (*e.g*., x-ray, cryo-EM, homology-modelling, or AlphaFold^83^)] will promote the discovery of drug candidates with distinctive mode of action. Furthermore, the logistics outlined in this work also provide a useful framework for similar data mining and descriptor approaches with respect to different pharmacological targets [*e.g.,* under-studied human/bacterial ABC transporters, G-protein coupled receptors (GPCRs), ion channels (ICs), solute carriers (SLCs; PANSLC, http://www.panslc.info) or tyrosine kinases (TKs)].

## Data Availability

The ABC_BPMDS is available without any restrictions under the http://www.zenodo.org^28^ URL (10.5281/zenodo.6384343). In addition, a detailed curation protocol including a GraphPad Prism file are provided under the https://www.zenodo.org^50^ URL (10.5281/zenodo.6405752). All information is also available on the http://www.panabc.info website and the use is free of charge.
